# Management of Bilateral Dermoid Cysts With Bilateral Torsion During the First Trimester of Pregnancy: A Rare Case Report

**DOI:** 10.7759/cureus.76992

**Published:** 2025-01-06

**Authors:** Poonam Lal, Swaroop R Nanda, Kumari Ruhi

**Affiliations:** 1 Department of Obstetrics and Gynaecology, Kurji Holy Family Hospital, Patna, IND

**Keywords:** 1st trimester, dermoid cysts, laparotomy, pregnancy, torsion

## Abstract

Dermoid tumors of the ovary in reproductive age can get complicated by torsion. We report a case in which bilateral dermoid cysts caused bilateral torsion at eight weeks of pregnancy, necessitating laparotomy as well as left oophorectomy and right cystectomy with a successful obstetric outcome. A 25-year-old G3P2 at eight weeks of gestation presented with severe lower abdominal pain in the left iliac fossa area. Ultrasonography (USG) and magnetic resonance imaging (MRI) revealed bilateral adnexal masses (9.4 cm in the right adnexa and 11 cm in the left adnexa), suggestive of bilateral large dermoid cysts. Failure of conservative management led to the diagnosis of ovarian torsion. Exploratory laparotomy was performed after consenting to oophorectomy. On laparotomy, a large left dermoid cyst was twisted three to four times over its pedicle. The whole ovary was replaced by a dermoid cyst, was discolored, and didn’t regain its color after detorsion. Therefore, the left oophorectomy and right cystectomy were done. The post-op period and pregnancy remained uneventful. The patient delivered a female baby vaginally at 39 weeks. Therefore, a high index of suspicion should be for the possibility of acute ovarian torsion in pregnancy with a large dermoid cyst or a bilateral tumor, even in the first trimester. Early surgical intervention in such cases can salvage the ovary and reduce the risk of complications for the mother and fetus.

## Introduction

The incidence of adnexal masses during pregnancy comprises 0.2-2% of all cases [1]. Dermoid cysts are the most common ovarian tumors in the childbearing age. The incidence of dermoid cysts is 0.03% of all pregnancies [2,3]. They are formed from germ cell layers and typically contain ectodermal tissues like skin, hair, and nails; mesodermal tissues like fat and muscle; and endodermic tissues [4].

They are bilateral in 10% of cases [5]. The complications of dermoid cysts during pregnancy are torsion, rupture, and infection [6]. Torsion is the most common complication of ovarian dermoid cysts, occurring in 3-15% of cases [7,8]. It occurs as the result of the ovarian pedicle partially or completely twisting at its axis or ovarian ligament [8]. The causes are young age, pregnancy, multiparity, large size, and the presence of bilateral dermoid. Surgical intervention is usually deferred till early in the second trimester due to fetal risk like miscarriage [2].

We report a rare case of bilateral dermoid cysts that caused bilateral torsion at eight weeks gestation and necessitated laparotomy as well as left oophorectomy and right ovarian cystectomy. The patient delivered a healthy baby at 39 weeks.

## Case presentation

A 25-year-old multipara G3P2L2 at eight weeks gestation presented on March 2, 2022, at Kurji Holy Family Hospital, Patna, India, with complaints of severe pain in the lower abdomen in both quadrants, more on the left side. She had mild nausea and vomiting. Her vitals were stable; her blood pressure (BP) was 110/70 mmHg, her pulse was 80 beats/min, and she was afebrile. On investigations, Hb was 8.5 gm/dL; total leukocyte count (TLC) was 6400/cm³; platelets were normal; viral markers (hepatitis B, HIV, and HCV) were negative; and blood sugar, liver function test (LFT), kidney function test (KFT), electrolytes, bleeding time (BT), clotting time (CT), and prothrombin time (PT) were normal.

Per the abdomen examination, there was an irregular palpable lump about 30 weeks uterine size. A lump was palpable in both iliac fossae (larger and more tender on the left side). The patient was put on conservative management. Ultrasonography (USG) on March 2, 2022, showed bilateral complex cystic adnexal masses, 9.4 × 8.4 cm in the right adnexa and 11 × 10 cm in the left adnexa, giving acoustic shadow and internal echoes suggestive of bilateral large dermoid cysts. There was no evidence of torsion, which was supported by findings of color Doppler. Magnetic resonance imaging (MRI) on March 4, 2022, also showed bilateral dermoid cysts with mild free fluid in the pelvis.

As the patient didn’t improve on conservative management, a provisional diagnosis of torsion was made. The fetal risk vs. maternal risk due to torsion was explained. Exploratory laparotomy was done after informed consent of oophorectomy.

**Figure 1:**
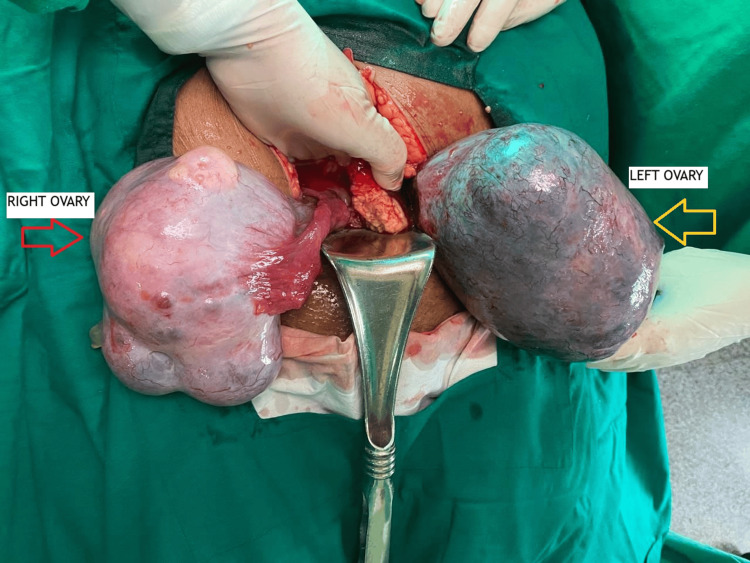
Intra-operative picture showing bilateral dermoid cysts with bilateral torsion and left ischemic ovary

**Figure 2:**
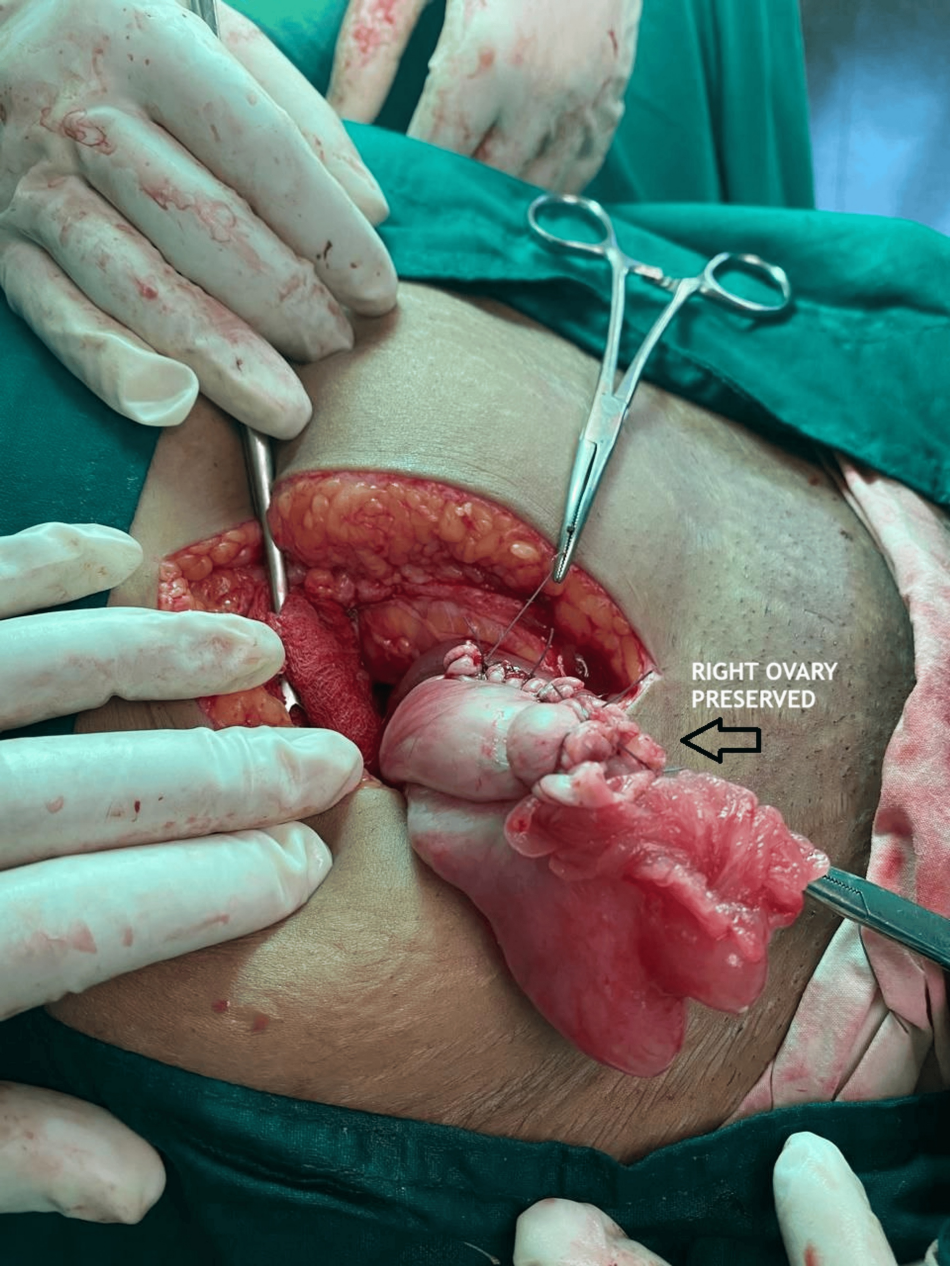
Intra-operative picture showing preserved right ovary (post cystectomy)

**Figure 3:**
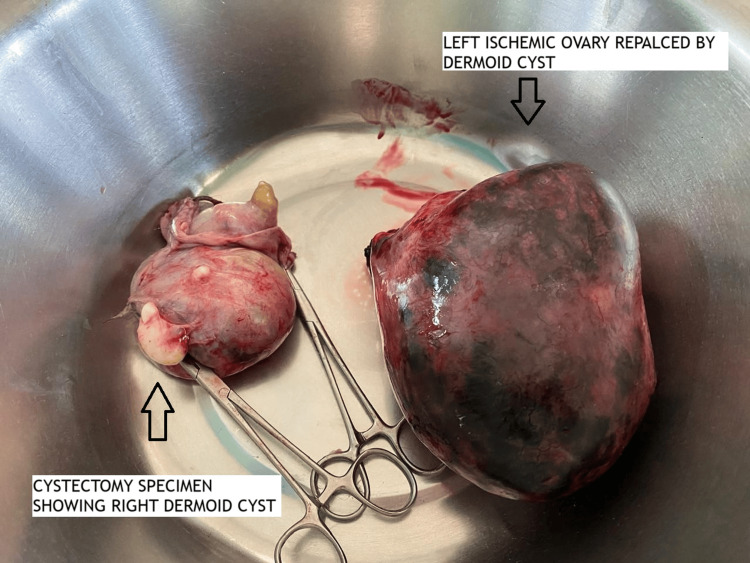
Retrieved right dermoid cyst and left oophorectomized specimen

**Figure 4:**
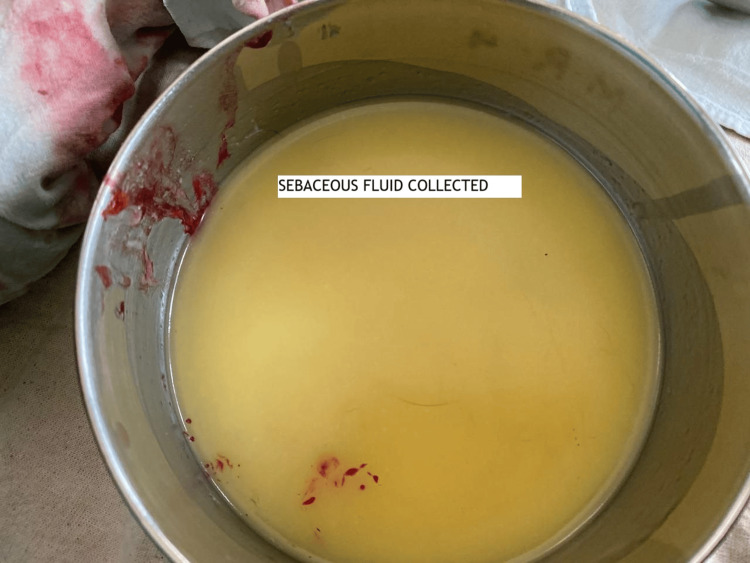
Sebaceous material collected from the cysts

On laparotomy, a large left dermoid cyst, 11 × 9 cm, was found twisted three to four times over its pedicle. The whole ovary was replaced by a black-colored dermoid cyst, and it didn’t regain its color in spite of detorsion (Figure 1). Cystectomy of the ischemic ovary was avoided as it invites significant bleeding and loss of ovarian tissue. Moreover, we couldn’t find significant ovarian tissue; therefore, a left oophorectomy was done. A large right dermoid cyst, 9 × 8 cm in size, was twisted two times over its pedicle with normal ovarian tissue (Figure 1); therefore, a right ovarian cystectomy was done to preserve the ovarian tissue as much as possible (Figure 2). The retrieved surgical specimens were sent for histopathological examination (Figures 3, 4). The histopathological report confirmed the presence of mature keratinized squamous cells and adipose tissue, suggestive of mature cystic teratoma of the ovary.

Blood loss was 120 mL, and one unit of packed RBC was transfused to maintain postoperative hemoglobin and improve postoperative recovery. Post-op USG was performed for fetal viability and was normal. Post-operative recovery was very good. The patient was discharged on the fifth day of surgery. The antenatal period remained uneventful. Growth scans were normal. The patient had a spontaneous vaginal delivery of a healthy female baby weighing 3 kg with a good APGAR score on October 6, 2022.

## Discussion

Ovarian torsion is the most common complication of dermoid cysts. Ovarian torsion is the complete or partial rotation of the ovary on its ligamentous support. Studies show that risk factors for ovarian torsion are ovarian mass >6 cm, multiparity, pregnancy, ovulation induction, and prior torsion [3,9]. Clinical symptoms of torsion are pelvic pain, adnexal mass, nausea, vomiting, fever, and vaginal bleeding [8].

On top of USG findings that point to a dermoid cyst, torsion is marked by an enlarged ovary due to venous and lymphatic congestion, as well as peripherally displaced follicles and a flipped ovary sign [10]. A positive whirlpool sign in the twisted vascular pedicle of the ovary is the most definitive sign of ovarian torsion [11]. The absence of blood flow in the twisted pedicle and the visualization of flow in the artery alone are predictive of the non-viability of the ovaries. Up to 20% of adnexal masses are thought to be insufficiently visible for thorough ultrasonography examination. MRI is the best modality for better characterization and assessment of these abnormalities. MRI is superior to transvaginal sonography in diagnosing torsion in pregnancy [12,13].

Researchers show that large dermoid tumors (mean diameter, 10.8 cm) are more likely to go into torsion. Bilateral dermoid cysts are more likely to torse.

A study done by Hoover and Jenkins on the management of adnexal mass concluded that surgical intervention in pregnancy is indicated for (1) asymptomatic patients with size >6 cm, (2) bilateral tumoral and multiparty, (3) suspicion of malignancy, and (4) torsion, rupture, or infection [14].

There remains a dilemma in management due to the risk of surgery and anesthesia versus the risk of a persistent mass. Surgery is deferred till the second trimester. Early prophylactic surgical intervention should be done, if possible, with laparoscopy for dermoid cysts between 5 and 10 cm [15].

When surgery is necessary, laparoscopy is safer (size, 5-10 cm) as it reduces pain, shortens stay, and decreases bleeding. Laparotomy may be more appropriate if ovarian tumors are larger (>10 cm) and have solid components [14].

Many researchers have reported cases of unilateral and bilateral ovarian cysts in the first and second trimesters, mostly with unilateral torsion. In most cases, laparoscopy has been done, but laparotomy is done if the dermoid cyst is >10 cm. Cystectomy and oophorectomy were decided according to the size and viability of the ovary [16-20].

We conducted a literature review of different case reports on dermoid cyst torsion in the first trimester of pregnancy, which is depicted in Table 1.

**Table 1:** Management of dermoid cyst in the first trimester by different researchers U/L, unilateral; B/L, bilateral

Researcher	Gestation	Bilateral/unilateral torsion	Size	Management
Gupta et al. (2023) [16]	5 weeks	U/L torsion	5.8 x 4 cm	Laparoscopic salpingo-oophorectomy
Surducki (2020) [17]	11 weeks	U/L torsion	5 x 4 cm	Laparoscopic salpingo-oophorectomy
Alleppanavar and Gomathy (2020) [18]	10 weeks	U/L torsion	10 x 5 cm	Laparotomy + oophorectomy
Özler et al. (2015) [19]	10 weeks	Bilateral dermoid with U/L torsion	6 x 5 cm	Laparoscopic cystectomy
Alalade and Abdelmagied (2010) [20]	8 weeks	U/L torsion	8 x 5 cm	Laparoscopic oophorectomy

Gupta et al. reported torsion of the right ovarian dermoid of 5.8 × 4 cm at four weeks of gestation and performed lap salpingo-oophorectomy [16]. Surducki reported right-sided torsion at 11 weeks of a 5 cm dermoid and did laparoscopic salpingo-oophorectomy [17]. Alleppanavar and Gomathy reported torsion of a right-sided dermoid of 10 × 5 cm size at 10 weeks, for which they performed laparotomy and oophorectomy [18]. Özler et al. reported a case of bilateral dermoid cysts in the first trimester with unilateral torsion, but the size was less than 10 cm, so they did a laparoscopic cystectomy [19]. Alalade and Abdelmagied reported torsion of a left dermoid cyst of 8 × 5 cm size at eight weeks and did laparoscopic oophorectomy [20].

Dhobale et al. (2023) [3] reported right twisted dermoid cysts of 14 × 10 cm size in the second trimester, and they did laparotomy and oophorectomy. 

Our case report is rare as it was large bilateral dermoid cysts >10 cm with bilateral torsion, which necessitated laparotomy in the first trimester as well as left oophorectomy and right cystectomy. The patient delivered a female baby vaginally at 39 weeks with a favorable APGAR score.

## Conclusions

High index suspicion should be there for the possibility of acute torsion in pregnant women with dermoid cysts >6 cm or bilateral tumors even in the first trimester. USG with Doppler examination of ovarian cysts and pedicles has high predictive value but also has high false-negative rates. MRI is superior in diagnosing torsion, but clinical suspicion of torsion should override the imaging alone.

Early intervention in such cases can salvage the ovary and reduce the risk of complications for the mother and fetus. The decision of cystectomy or oophorectomy depends on the presence or absence of ischemia, the size of cysts, the type of cysts, and the patient’s choice.
